# Building a Morphogen Gradient without Diffusion in a Growing Tissue

**DOI:** 10.1371/journal.pone.0012857

**Published:** 2010-09-30

**Authors:** Rebecca H. Chisholm, Barry D. Hughes, Kerry A. Landman

**Affiliations:** Department of Mathematics and Statistics, University of Melbourne, Victoria, Australia; University of Arizona, United States of America

## Abstract

In many developmental systems, spatial pattern arises from morphogen gradients, which provide positional information for cells to determine their fate. Typically, diffusion is thought to be the mechanism responsible for building a morphogen gradient. An alternative mechanism is investigated here. Using mathematical modeling, we demonstrate how a non-diffusive morphogen concentration gradient can develop in axially growing tissue systems, where growth is due to cell proliferation only. Two distinct cases are considered: in the first, all cell proliferation occurs in a localized zone where active transcription of a morphogen-producing gene occurs, and in the second, cell proliferation is uniformly distributed throughout the tissue, occurring in both the active transcription zone and beyond. A cell containing morphogen mRNA produces the morphogen protein, hence any gradient in mRNA transcripts translates into a corresponding morphogen protein gradient. Proliferation-driven growth gives rise to both advection (the transport term) and dilution (a reaction term). These two key mechanisms determine the resultant mRNA transcript distribution. Using the full range of uniform initial conditions, we show that advection and dilution due to cell proliferation are, in general, sufficient for morphogen gradient formation for both types of axially growing systems. In particular, mRNA transcript degradation is not necessary for gradient formation; it is only necessary with localized proliferation for one special value of the initial concentration. Furthermore, the morphogen concentration decreases with distance away from the transcription zone, except in the case of localized proliferation with the initial concentration sufficiently large, when the concentration can either increase with distance from the transcription zone or sustain a local minimum. In both localized and uniformly distributed proliferation, in order for a concentration gradient to form across the whole domain, transcription must occur in a zone equal to the initial domain size; otherwise, it will only form across part of the tissue.

## Introduction

Pattern formation occurs in many developmental processes, including somitogenesis [1]–[3], limb bud development [4]–[6] and other processes [7]–[11]. In many cases, a chemical morphogen, via the existence of a morphogen gradient, determines cell fate and the resulting spatial patterns. Consequently, the formation of morphogen gradients has been the subject of many studies [12]–[18].

To understand how a morphogen gradient forms in a specific tissue system, it is necessary to understand the mechanisms behind morphogen production (the source), cell interaction with and response to the morphogen, and morphogen transport throughout the system. The physical nature of the system is also vital to understanding gradient formation. For example, if the system is undergoing growth, then both cells and morphogen can be actively transported through the tissue. Similarly, if the cells responsible for producing morphogen can divide or are free to move around, then this will also affect morphogen transport. Cell arrangement, cell density and the form of the extracellular matrix will determine whether the morphogen is capable of long-range diffusion. Also, multiple morphogen gradients may interact and regulate each other in the same system [16].

There are numerous arguments for [8], [15], [19], [20] and against [16], [21] diffusion as a mechanism contributing to morphogen transport. Despite these, popular models of morphogen gradient formation [15], [18], [22] and mathematical models of developmental processes [23], [24] usually include a diffusive mechanism for morphogen transport. There are several alternatives to diffusion as a mechanism for morphogen transport. One of these is transcytosis, where morphogen is relayed internally between cells. In this type of vesicle-mediated transport, neighbouring cells undergo a cycle of endocytosis and re-secretion of receptor-bound morphogen [14]. Another mechanism is morphogen transport via cytonemes, which are actin-based cellular extensions which may extend for distances many times the diameter of the cells [25]. Here we show that there is a third alternative which is possible if the tissue system is undergoing axial growth via cell proliferation.

Using mathematical models, we investigate morphogen gradient formation in two types of axially growing systems, where growth is due to cell proliferation only. Proliferation-driven growth gives rise to both advection (the transport mechanism) and dilution (a reaction mechanism). Advection is a form of bulk movement analogous to fluid flow. Here we discard diffusion, the usual transport mechanism associated with a morphogen gradient. Furthermore, we show that advection and dilution, as a result of cell proliferation, are sufficient to set up a morphogen gradient under certain conditions. In particular, we show that diffusion is not necessary to achieve gradient formation. We also show that morphogen degradation is not necessary except in a special case.

**Figure 1:**
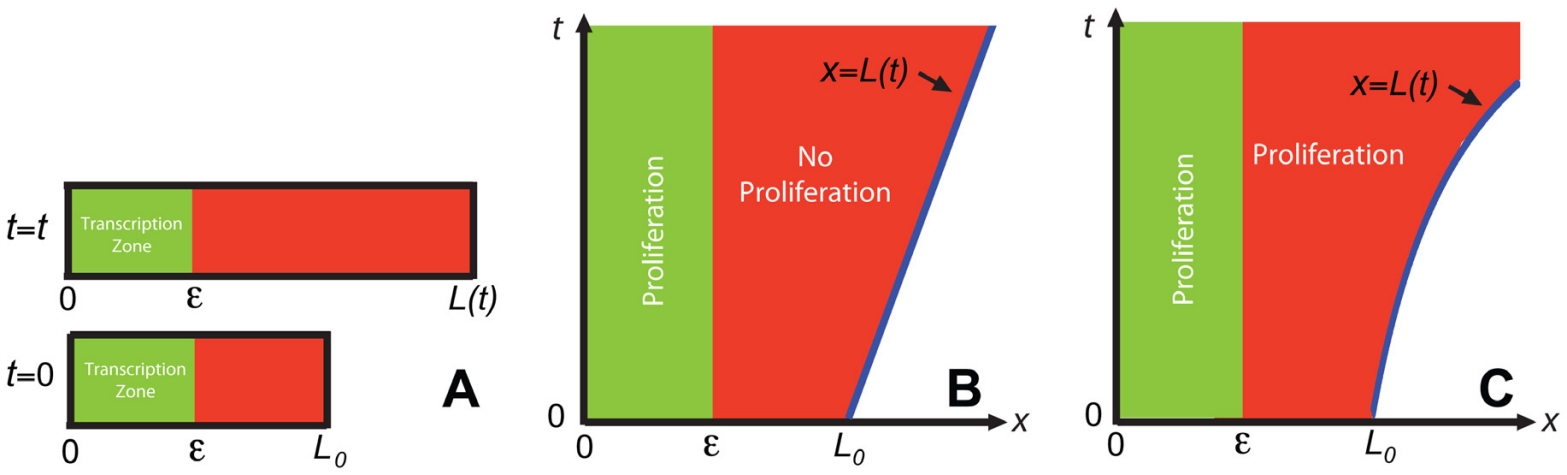
Schematic diagrams of transcription zone (green) and zones of proliferation. (A) Transcription zone in axially growing tissue at 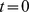 and at a later time 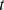. (B and C) Space (horizontal axis) versus time (vertical axis) diagram of transcription zone and zones of proliferation in the growing tissue. (B) Localized zone of cell proliferation. (C) Uniformly distributed cell proliferation.

We consider a localized zone of active morphogen gene transcription in these systems. In this zone, cells divide into two daughter cells, causing cells to be actively transported out of the transcription zone. We assume that the zone of transcription remains a constant length, 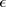, for all time (Figure 1A). This idea is a simplification of the situation that Pfeiffer and coworkers [16] observed in the formation of the wingless (wg) gradient in *Drosophila* embryos. In *Drosophila*, cells that transcribe *wg* are found in stripes of cells adjacent to a source of Hedgehog protein. These cells divide and are subsequently actively transported away from the Hedgehog source. When the concentration of Hedgehog around a cell has fallen below a threshold, they stop transcribing *wg*. Here we assume a threshold length (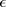) of the transcription zone.

In our models cells outside of the transcription zone cannot continue active gene transcription. However, if a cell contains morphogen mRNA, it produces the morphogen protein. In this way, if there is a gradient of mRNA within the system, this will translate into a morphogen protein gradient. When a cell divides into two new daughter cells, the two new cells are assumed to inherit an equal share of their parent cell's mRNA transcripts. Therefore, cell division also results in morphogen mRNA dilution and consequently morphogen protein dilution.

We assume that there is a direct correlation between mRNA transcript concentration and the concentration of the morphogen protein. This is a reasonable assumption given that the morphogen protein cannot diffuse. In doing so, we can model the concentration of the morphogen's mRNA transcripts in the system and equate the corresponding mRNA transcript concentration profile to that of the morphogen protein concentration profile. A key idea in our model is that morphogen mRNA resides within cells and the cells contain varying amounts of morphogen mRNA – this gives rise to a spatial distribution of morphogen protein within the tissue, without the need for extracellular morphogen protein diffusion.

A similar mechanism for gradient formation has been suggested in a system where there is overlap between the morphogen protein gradient and morphogen mRNA gradient. Dubrulle and Pourquié [3] found that the gradient of FGF8 protein, present during somitogenesis in chick and mouse embryos, results from a gradient of FGF8 mRNA transcripts. They suggested that the formation of this transcript gradient, and thus the morphogen protein gradient, is due to the combination of a localized transcription zone, mRNA degradation and cell division in the transcription zone. Here, we also show that mRNA degradation is generally not necessary for morphogen gradient formation in our model systems – this is contrary to the suggestion of Dubrulle and Pourquié [3].

## Results

### Mathematical model

We model the axially elongating tissue in one continuous spatial dimension, 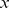, and in continuous time, 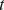. Initially, the tissue has length 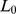, and the tissue length at time 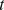 is 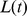. The form of 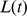 depends on the distribution of cell divisions within the tissue. The morphogen mRNA transcript concentration, 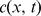, satisfies the principle of the conservation of mass, resulting in a partial differential equation on an elongating tissue 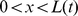,
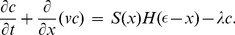
(1)Cell proliferation produces axial elongation, inducing an advective transport term, 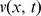, which depends on the distribution of cell divisions [12]. The transcription zone is the region 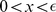, and its mRNA source has strength 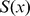; the Heaviside step function, 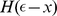, which takes the values 0 and 1 for 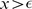 and 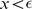, respectively, is used to turn the source off outside of the transcription zone (Figure 1A). We present results for a spatially invariant source where 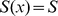, a constant. A morphogen mRNA transcript degradation term, 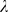, is included.

Equation (1) can be rewritten into a form which highlights the two key mechanisms arising from proliferation-driven growth. Expanding the second term in Equation (1) gives
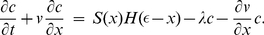
(2)The term 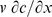 is the advective transport term and the term 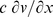 is the dilution term.

We assume that initially there is uniform morphogen mRNA transcript concentration 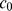 present in the system, that is, 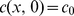, where 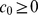. This means that any gradients that form are the result of proliferation-driven growth alone. Here morphogen mRNA cannot leave the growing tissue, giving rise to no–flux boundary conditions (which are automatically satisfied in our model):
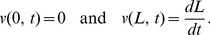
(3)


Assuming that there is a direct correlation between morphogen mRNA transcript concentration and the morphogen protein concentration, our results for generating a morphogen mRNA gradient can be translated into a corresponding morphogen protein gradient.

### Cell proliferation restricted to a localized zone

Consider the case where the zone of transcription is the only zone of cell proliferation (Figure 1B). Cells within this zone divide at a rate 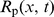 per unit length. Because the zone of transcription is a constant length, 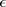, these cell divisions will result in cells being ‘pushed out’ of the zone at a rate 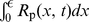. We present results for a spatially constant proliferation rate, 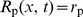, giving 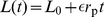. The convective transport term 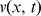 takes a different form inside and outside of the transcription zone:
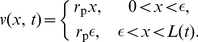
(4)Note that from Equation (2), there is a dilution term in the transcription zone only, and it has strength 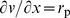. The solution to Equation (1) with 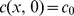 is
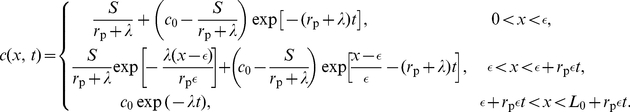
(5)This solution is investigated in detail below, separating the two cases 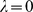 and 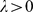. Our results will demonstrate that a morphogen mRNA gradient is always established across the region 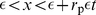, except for the special case when 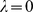 and 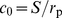. However, the properties of the resulting mRNA concentration distribution may vary greatly.

It is useful to define the equilibrium concentration in the transcription zone, namely
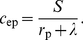
(6)This is the concentration in the transcription zone which produces a balance between the constant source term (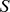) and two possible sink terms (dilution and degradation, 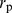 and 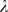 respectively).

### No transcript degradation (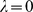)

Addressing first the case 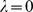, the solution for the concentration simplifies to
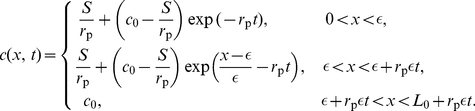
(7)Except for the special case 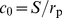, the concentration 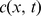 varies with position 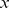 outside the transcription zone in the region 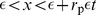 at each fixed time 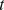. This is contrary to the suggestion of Dubrulle and Pourquié [3], namely that mRNA degradation (corresponding here to 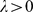) is necessary for gradient formation in a similarly growing system. For our model, 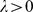 is required to produce a concentration gradient only for the special case when 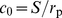.

**Figure 2:**
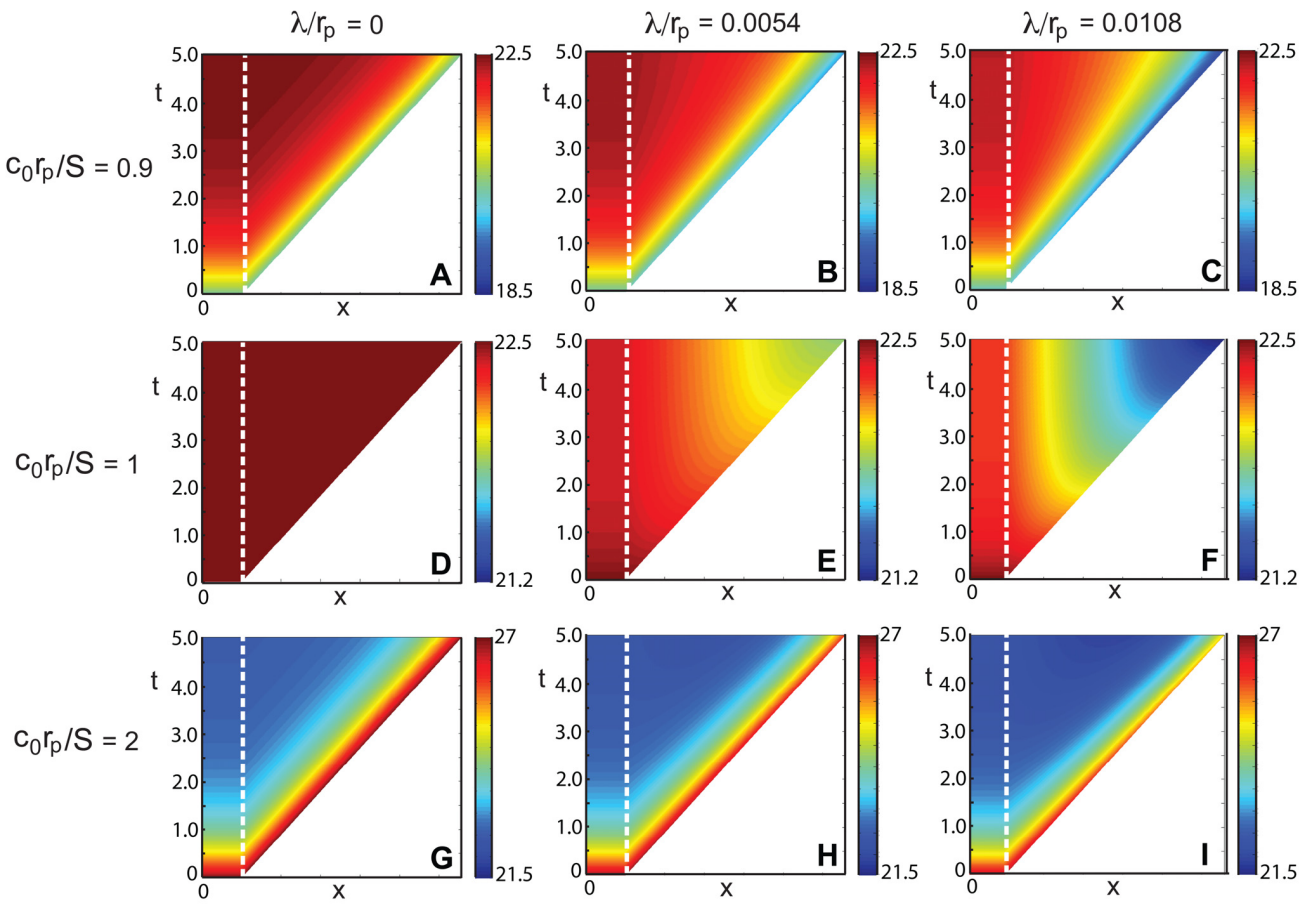
Space–time plots of the morphogen mRNA concentration with a localized zone of cell proliferation as a function of distance 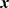 (horizontal axis) and time 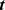 (vertical axis), for various values of 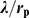 and 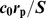. The color indicates the mRNA concentration 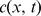 at that position and time point, as given by the color bar. Here 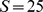, 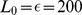 and 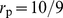. In each plot, the dashed line indicates the extent of the transcription zone (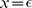).

**Figure 3:**
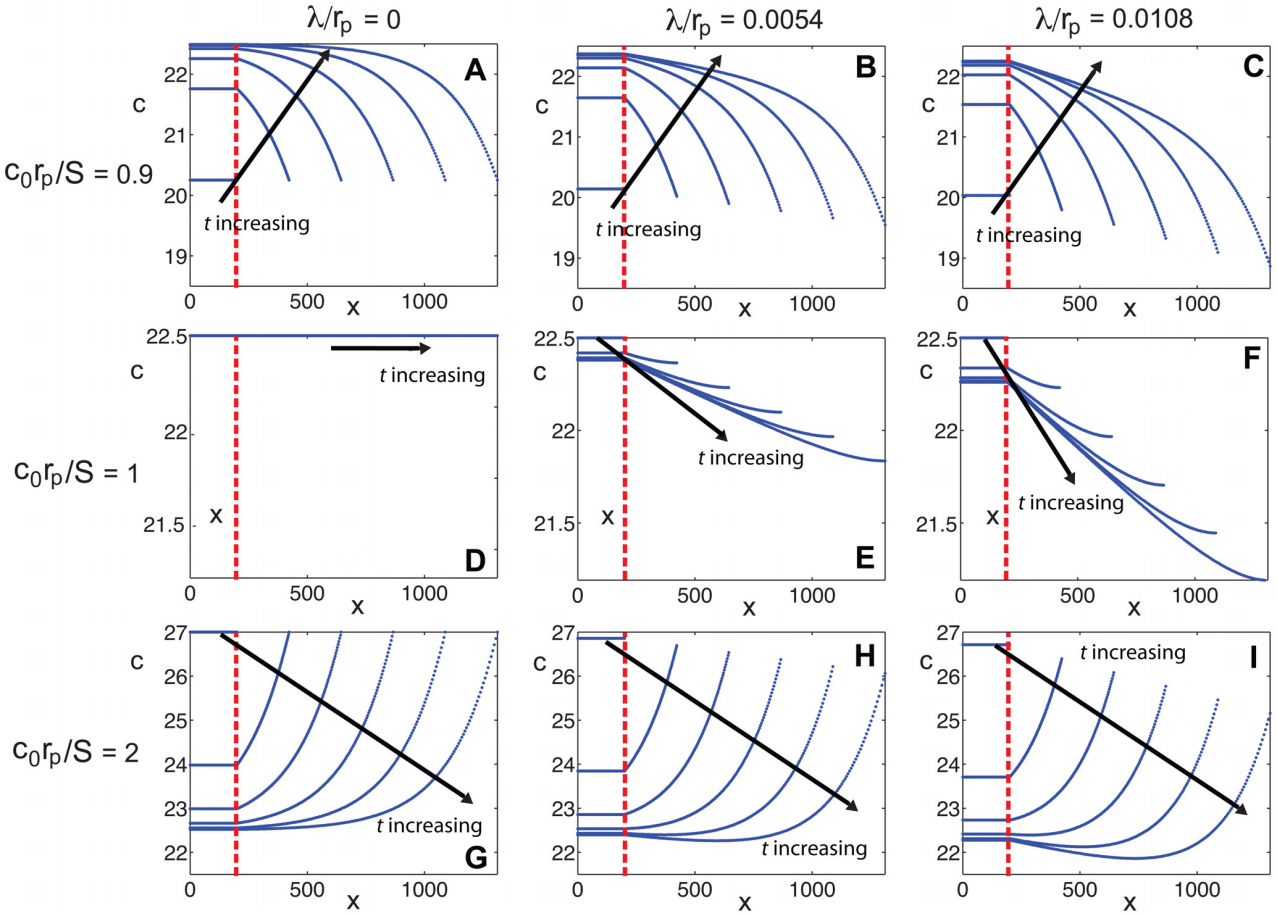
Morphogen mRNA concentration with a localized zone of cell proliferation as a function of distance 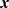 at different times for various values of 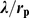 and 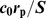. The concentration profiles are for times 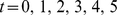, the arrow indicating the direction of increasing 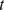. Here 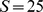, 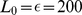 and 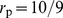. In each plot, the dashed line indicates the extent of the transcription zone (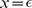).

For the case when 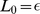, space–time plots of the concentration (Figure 2 left column) given by Equation (7) clearly display how the morphogen mRNA concentration behaves differently outside the zone of transcription, for varying values of the ratio 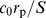. Plots of the concentration at different times with the same parameter values (Figure 3 left column) also illustrate this result. The importance of the relative magnitudes of 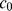 and 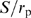 to the nature of the concentration gradient is readily apparent. For 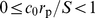, the concentration is a decreasing function of 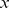 throughout the interval 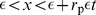, while if 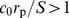 the concentration increases with distance from the transcription zone. Note that for the case 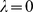, 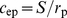, so 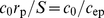.

**Figure 4:**
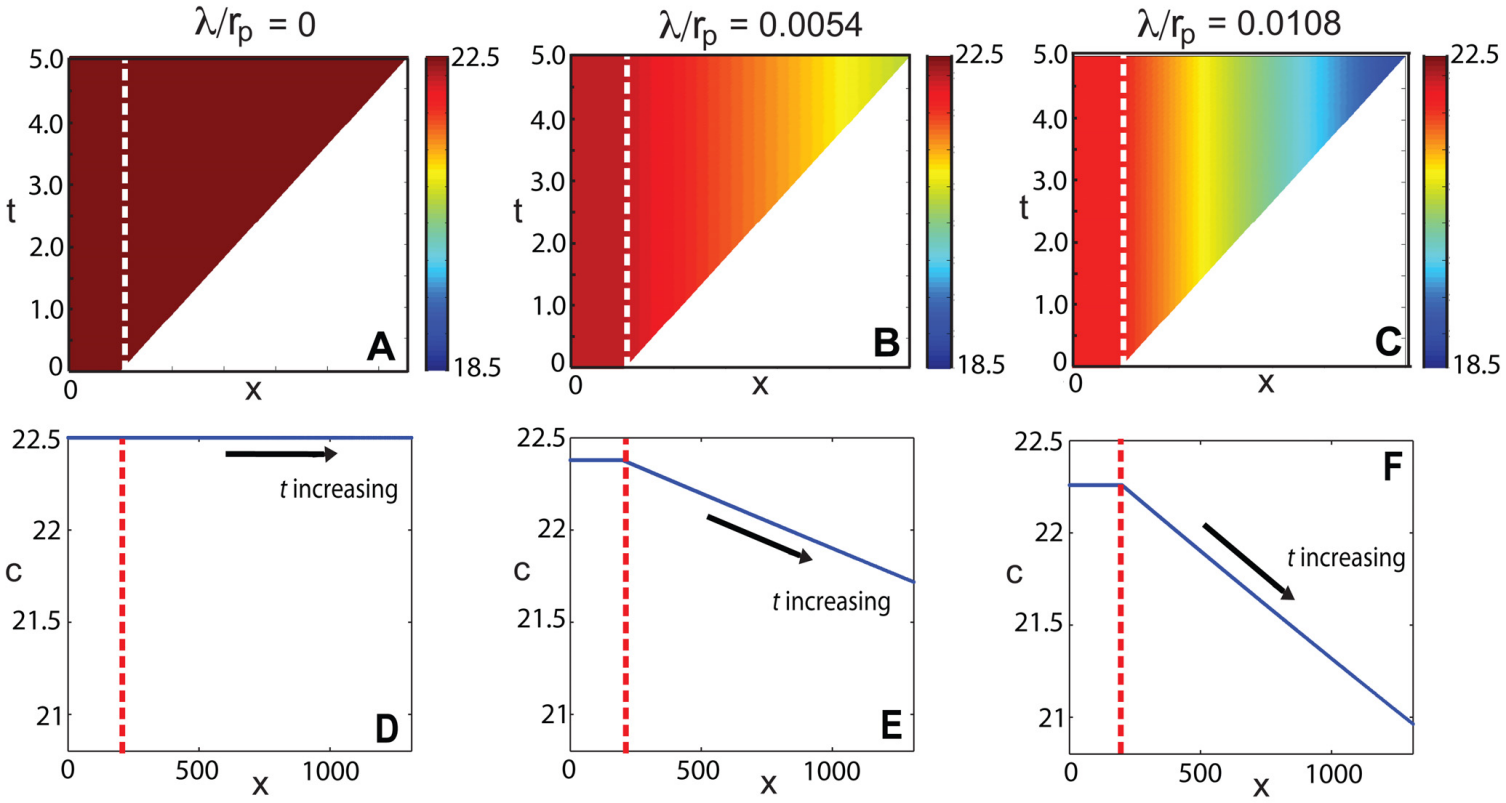
Morphogen mRNA concentration with a localized zone of cell proliferation for 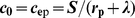 for various values of 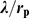. (A–C) Space–time plots of the morphogen mRNA concentration as a function of distance 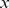 (horizontal axis) and time 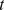 (vertical axis). The color indicates the mRNA concentration 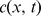 at that position and time point, as given by the color bar. (D–F) Plots of the concentration as a function of distance 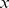 at times 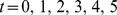, the arrow indicating the direction of increasing 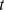. Here 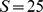, 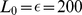 and 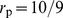. Here 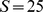, 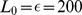 and 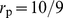. In each plot, the dashed line indicates the extent of the transcription zone (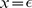).

Note also that for fixed position 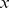 within 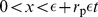, if 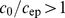, the concentration will decrease with time, but if 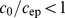, the concentration will increase with time (Figure 3 left column). When the initial concentration equals the equilibrium concentration, 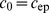, the distribution of the morphogen mRNA concentration will remain constant for all time, for each position within the growing tissue (Figure 3D, Figure 4 left column).

### With transcript degradation (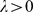)

If 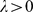 there is always a morphogen gradient in the region 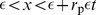. For the case when 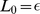, space–time plots of the concentration (Figure 2 center and right columns) given by Equation (5), clearly display how the morphogen mRNA concentration behaves differently outside the zone of transcription, for varying values of 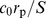 and 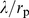. Plots of the concentration at different times with the same parameter values (Figure 3 center and right columns) also illustrate this result. In fact, the figures illustrate that the concentration can either decrease, increase, or have a local minimum, as a function of distance from the transcription zone, depending on 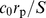 and the time of observation.

Although for 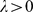 we have 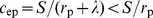, it is the size of the parameter 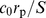 (rather than 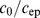) that influences the nature of the gradient that forms. When 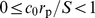, the mRNA concentration given by Equation (5) is a decreasing function of the distance from the transcription zone, throughout the interval 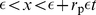. However, when 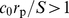, the qualitative behaviour of 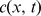 changes with time. For sufficiently early times 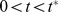, 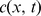 is an increasing function of 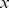 for 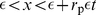, so a morphogen mRNA gradient is established, but in the opposite direction to the small 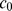 case. For later times 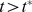, the concentration first decreases to a local minimum at 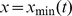 and thereafter increases with 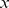. Here the critical time 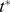 and position 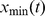 are given by
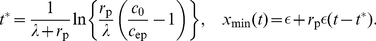
(8)


For fixed position 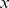 within 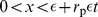, the concentration will decrease or increase with time depending on 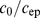, rather than 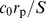. If 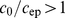, the concentration will decrease with time, but if 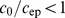, the concentration will increase with time. When the initial concentration equals the equilibrium concentration, 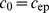, the distribution of the morphogen mRNA concentration will remain constant for all time, for each position within the growing tissue, as illustrated in the center and right columns of Figure 4.

### Concavity of the transcript concentration profile

A further characterization of the shape of the concentration profile 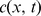 in the interval 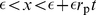 comes from the study of the second 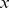-derivative 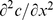. When it is positive, then the concentration 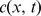 is concave up; alternatively when it is negative, then 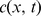 is concave down. The key result is that the concavity of concentration profiles depends on the size of 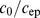.

When 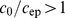 and all 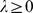, 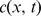 is concave up throughout the interval 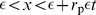. For the chosen parameter values, the center and bottom rows of Figure 3 illustrate this case. When 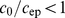 and 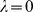, 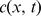 is always concave down. However, when 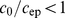 and 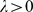, the concavity can change over time. If 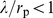 and 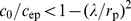 then, for sufficiently early times 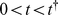, the concentration profile is concave down over the whole interval. For later times 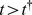, the profile is concave up at the left endpoint, contains an inflection point at 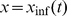 and thereafter is concave down. Here the time 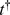 and position 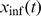 are given by
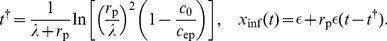
(9)For the chosen parameter values, Figure 3 top row illustrates this case – the inflection point has fully evolved in Figure 3C at 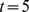.

### Cell proliferation uniformly distributed

Now consider the case where cell proliferation is distributed uniformly throughout the tissue (Figure 1C). All cells can proliferate and at a rate 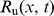 per unit length. We present results for a spatially constant proliferation rate, 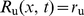, giving a differential equation describing the length of the tissue:
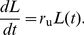
(10)Therefore, the tissue now grows exponentially as 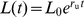. The appropriate convective transport term is given by
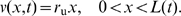
(11)Note that from Equation (2), there is a dilution term throughout the whole tissue, with strength 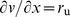. The solution to Equation (1) with 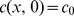 is now
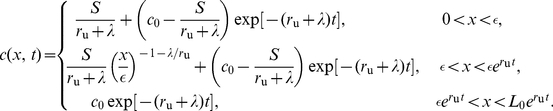
(12)In the interval 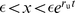, the concentration given by Equation (12) is decreasing as a negative power of distance 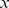 from the source for each time 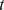. This is true for all 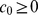 and all 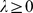. Therefore, a morphogen mRNA gradient is established in 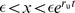. In particular, the mRNA concentration gradient forms even if mRNA does not degrade (case 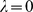).

**Figure 5:**
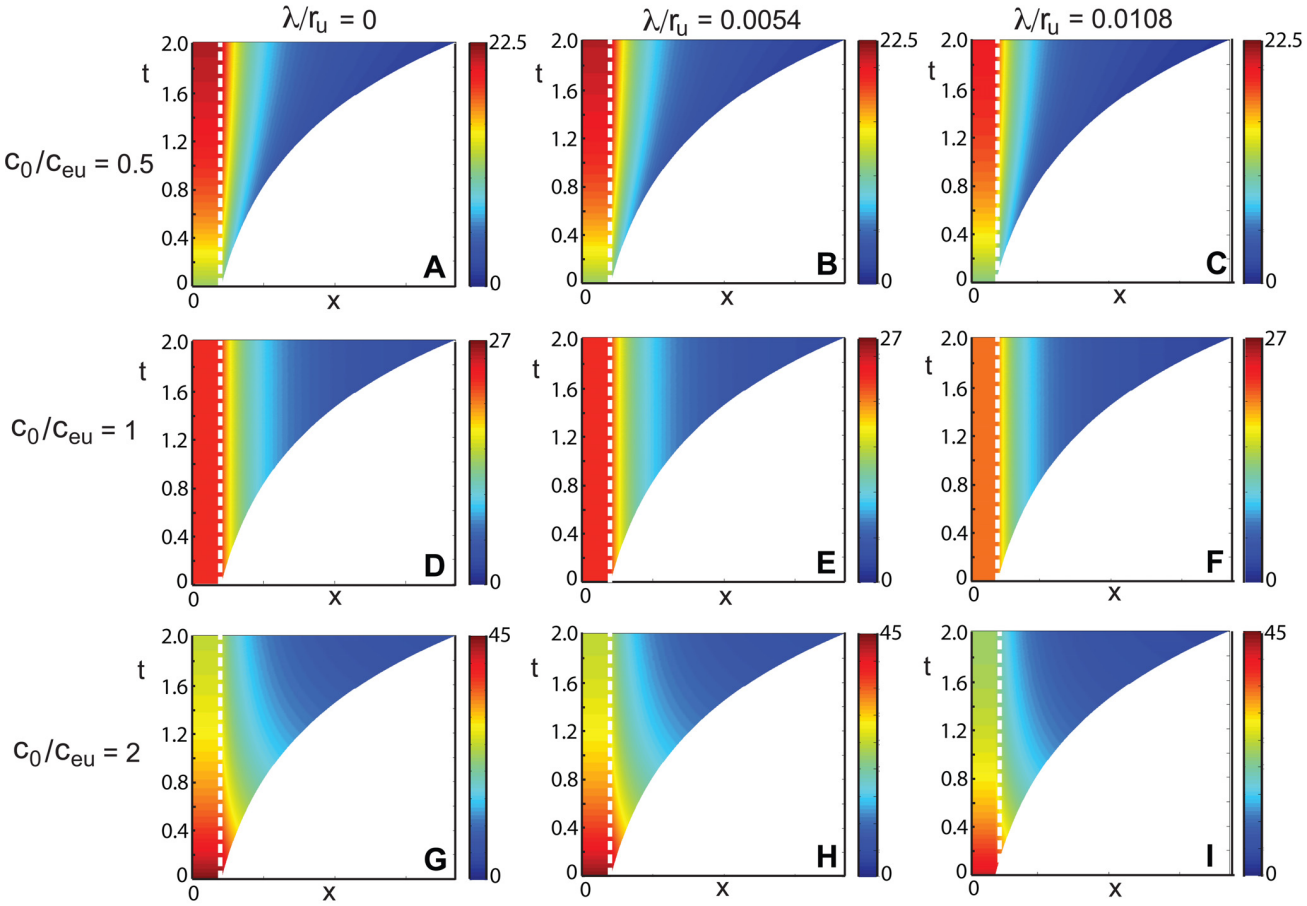
Space–time plots of the morphogen mRNA concentration with uniformly distributed cell proliferation as a function of distance 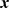 (horizontal axis) and time 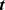 (vertical axis), for various values of 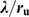 and 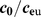. The color indicates the mRNA concentration 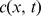 at that position and time point, as given by the color bar. Here 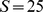, 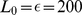 and 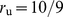. In each plot, the dashed line indicates the extent of the transcription zone (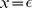).

**Figure 6:**
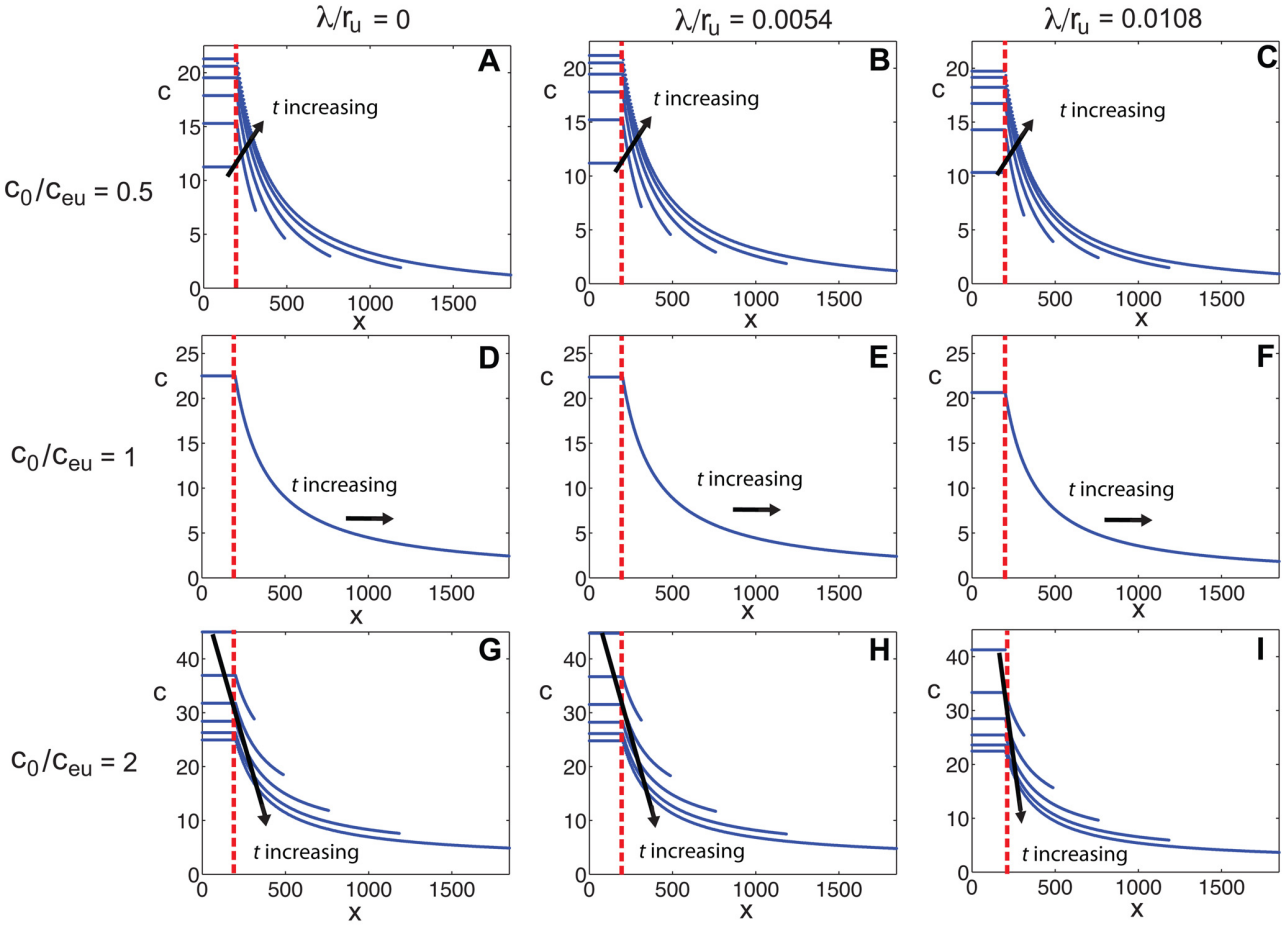
Morphogen mRNA concentration with uniformly distributed cell proliferation as a function of distance 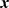 at different times for various values of 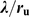 and 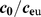. The concentration profiles are for times 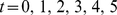, the arrow indicating the direction of increasing 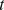. Here 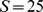, 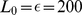 and 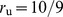. In each plot, the dashed line indicates the extent of the transcription zone (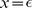).

It is useful to define the equilibrium concentration in the transcription zone, namely
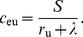
(13)For the case when 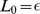, space–time plots of the concentration (Figure 5A–I) and concentration plots at different times (Figure 6A–I) for various values of 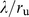 and 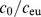 clearly display a decreasing morphogen mRNA concentration outside the zone of transcription. It is interesting to note that the shape of the concentration profiles is different from the localized proliferation case – here it is always concave upwards (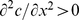). In particular, the concavity does not depend on the relative size of 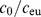. Again, at a fixed position 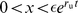, the concentration increases with time if 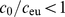, and the concentration decreases with time if 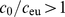. When the initial concentration equals the equilibrium concentration, 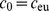, the distribution of the morphogen mRNA concentration will remain constant for all time, for each position within the growing tissue.

## Discussion

Using mathematical models, we have investigated the formation of a morphogen mRNA gradient, in the absence of diffusion, in a system undergoing axial elongation via cell proliferation, and where the morphogen protein gradient is correlated with its corresponding mRNA gradient. We focused on two different modes of axial elongation — elongation via a localized source of cells, where all cell proliferations are restricted to a localized zone at one end of the tissue where active transcription of the morphogen–producing gene occurs, and elongation via cell divisions distributed throughout the tissue. Both of these proliferation types occur during axial elongation in different animals groups – the first in annelids [26] and the second in arthropods and onychophorans [27], [28].

Other modes of elongation are theoretically possible – for example one where cell proliferation is restricted to a subset of the transcription zone. Analysis for this case shows that for sufficiently early times, a gradient will not form in that part of the transcription zone which is non-proliferative, but at later times, a gradient forms throughout the tissue (if 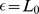) and is similar to that found for the localized proliferation case. Similar results occur in other variations of these models. In general, a model of an axially elongating tissue will give qualitatively similar results as long as there is relative bulk movement and mRNA dilution due to cell proliferation within the growing tissue and an appropriate initial concentration of morphogen mRNA in the transcription zone. Our model supports proliferation-driven axial growth as a means of establishing a morphogen concentration gradient without the need for diffusion or mRNA degradation (except for one special case). In both models, mRNA dilution and advection due to cell divisions is sufficient for morphogen gradient formation.

For the gradient to be established across the whole region outside of the transcription zone (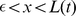), the length of the transcription zone must be equal to the initial length of the tissue, 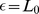. If 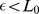, then a part of the system will never form a concentration gradient from a constant initial concentration. In a biological context, the assumption that 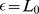 means that all cells initially present in the tissue are actively transcribing the morphogen gene and consequently that the ancestry of any cell present in the tissue at any time can be traced back to the transcription zone.

Our results imply that a morphogen gradient can arise in a growing system, even when morphogen protein diffusion is not possible and without morphogen mRNA degradation (except in one special case). This means that morphogen gradients can form in systems where previously it may have been thought impossible. For example, in large, highly cellularised systems, such as a developing onychophoran embryo, it may seem unlikely that a morphogen protein can diffuse the entire length of the embryo, given its size and the fact that the highly cellularised environment would be unable to support diffusion. However, our work shows that such a system can still theoretically form a morphogen concentration gradient. Interestingly, the morphogen gradient does not always have the highest concentration adjacent to the transcription zone. Indeed, for the case of localized proliferation when the initial concentration is sufficiently large, the concentration can either increase with distance from the transcription zone or sustain a local minimum. For all other cases, the morphogen concentration decreases with distance away from the transcription zone.

An interesting question arises from our analysis. Is it possible to distinguish between localized and global proliferation from knowledge of the morphogen distribution? An evaluation of our concentration profiles provides an answer. (i) If the concentration increases away from the transcription zone, or (ii) if the concentration decreases away from the transcription zone and the profiles are concave down sufficiently far from the transcription zone, or (iii) if the concentration does not change in time and decreases exponentially with 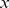, then the proliferation is localized. In contrast, (i) if the concentration decreases away from the transcription zone, the profiles are concave up and the concentration at a fixed position increases with time, or (ii) if the concentration does not change in time and decreases as a negative power in 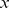, then the proliferation is uniformly distributed. Note that some of these criteria rely on multiple profiles at different times, whereas others just rely on a single profile at one time point. The proliferation extent cannot be resolved when the concentration profile has the following properties: the concentration decreases away from the transcription zone, the profiles are concave up and the concentration at a fixed position decreases with time (Figure 3E, 3F and Figure 6G, 6H, 6I). Our results therefore suggest that there are circumstances where it is possible to distinguish between localized and global proliferation from the morphogen gradient which has arisen through proliferation-driven transport, rather than diffusive processes.

## Materials and Methods

### Solution to partial differential equations

For the case of localized proliferation, the partial differential equation (1) with velocity given by (4) was solved using the method of characteristics [29]. For the case of distributed proliferation, the partial differential equation (1) with velocity given by (11) was solved by changing variables from 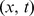 to 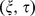 where 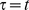 and 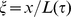. This resulted in a first order, linear ordinary differential equation, which was solved using the standard integrating factor method. The results were plotted using MATLAB software (MathWorks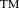).

The concavity results for localized proliferation system are obtained by evaluating 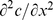 from Equation (5), and evaluating at the endpoints 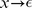 and 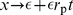, as
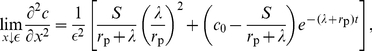
(14)

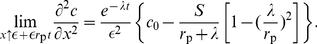
(15)


### Selection of parameter values

To produce the sample concentration profiles, parameter values consistent with experimental evidence for the vertebrate somitogenesis FGF8 gradient were chosen. Currently there are no measurements for invertebrates.

Gomez and coworkers [30] reported the somite size ranges between 75–175 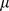
m and somitogenesis period of approximately 90 minutes in chick embryos. A consistent growth rate is then 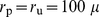
m
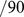
min. This ratio is of the same order as other vertebrate species that undergo somitogenesis includinging zebrafish (1.3 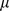
m
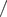
min), corn snakes (1 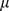
m
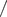
min) and mice (1.2 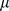
m
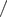
min) [30].

Dubrulle and Pourquié [31] estimated the half life of mRNA transcripts to be several hours. Using a half life of 2 hours, we estimated the mRNA degradation rate as 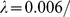
min.

Consistent with the somitogenesis model of Tiedemann and coworkers [32], the production rate of the mRNA transcripts was taken as 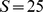 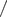
min. Also consistent with measurements of the tail bud and primitive streak in mouse embryos undergoing somitogenesis [33], we took the length of the transcription zone to be 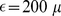
m.
